# The Heart-Brain Connection Begets Cardiovascular Psychiatry and Neurology

**DOI:** 10.1155/2009/546737

**Published:** 2009-01-28

**Authors:** Hari Manev

**Affiliations:** The Psychiatric Institute, Department of Psychiatry, University of Illinois at Chicago, Chicago, IL 60612, USA

In many Asian languages, the same ancient letter/symbol is used for *heart* and *mind*. For the last couple of millennia, physicians and scientists across different civilizations have posited close links between the brain and heart, even arguing that the site of intelligence and emotions is in one of these two organs. Through an integrative empirical approach in modern biology/physiology and behavioral sciences, much has since been learned to confirm the anatomical and functional links between the brain and the heart. Arguably, the ultimate applicability of this knowledge will advance our health and improve disease treatment.

Modern medicine is characterized by a high degree of specialization, and the heart-brain connection is typically considered from the point of view of a particular medical specialty. Hence, focusing on the brain, for example, in neurology, cardiovascular involvement is critical in certain pathologies such as stroke and vascular dementia. In cardiology, on the other hand, the influence of the brain becomes clearly apparent in “the broken heart syndrome” (also known as acute stress cardiomyopathy). However, recent epidemiological studies point to new associations that typically present as co-occurring pathologies of both the brain and the heart. A case in point is the association between depression and coronary heart disease. Such co-occurrences have stimulated research into possible novel mechanisms that could be targeted to treat these complex cardiovascular/brain disorders.

At least three scenarios could be at play in these illnesses: (i) the primary pathological mechanism in the nervous system triggers a cardiovascular pathology by disrupting physiological links between the two systems (hence, the term “psychogenic”cardiovascular disease), (ii) the primary pathological mechanism in the cardiovascular system triggers a nervous system dysfunction (e.g., atherosclerosis leading to ischemic conditions causes subsequent cognitive impairment), and (iii) the primary pathology is in a biological mechanism that is normally operative in both the nervous and the cardiovascular systems, thus causing the co-occurrence of pathologies (i.e., the co-occurring pathologies share a pathobiological mechanism but do not necessarily cause each other).

To be successful, research in co-occurring cardiovascular and brain disorders needs contributions from multiple medical specialties, including psychiatry, neurology, medicine, and cardiology. It should encompass clinical and basic research as well as the development of therapeutic approaches. The purpose of Cardiovascular Psychiatry and Neurology is to provide a platform for the latest research and for timely and expert reviews and comments in the emerging field of cardiovascular psychiatry and neurology. Although the term *cardiovascular psychiatry and neurology* is introduced here for the first time, retrieving publications from PubMed using either *cardiovascular psychiatry* or *cardiovascular neurology* generates relevant information (oftentimes because the terms *psychiatry* or *neurology* appear in the tile of a journal publishing the work). A quick survey of PubMed for the publication period from 1940 to 2008 (Table 1) revealed, not surprisingly, that the number of items per search term has increased dramatically over the years. Whereas the term *cardiovascular* showed a sharp increase in the early sixties, both *neurology* and *psychiatry* “exploded” in the early nineties. Even though the term *cardiovascular psychiatry* appeared earlier than *cardiovascular neurology*, the later term has become more prevalent since the early sixties (Table 1). Normalizing the data, that is, by expressing *cardiovascular psychiatry* as a percentage of corresponding *psychiatry* and *cardiovascular neurology* as a percentage of the corresponding *neurology* (Figure 1), showed that *cardiovascular neurology* rose sharply in the early sixties and has remained at about 5% of all *neurology*-related items. On the other hand, *cardiovascular psychiatry* had been below 1% of all *psychiatry*-related items until the early eighties, but has gradually increased. Possibly, the ischemia-related brain disorders have contributed to the relatively high presence of *cardiovascular neurology* and the relatively recent revelation of an unsuspected association of cardiovascular disorders with mood disorders might have contributed to the increase in *cardiovascular psychiatry*. The journal Cardiovascular Psychiatry and Neurology was created to provide for the first time a unified forum to consider the physiological and pathological interactions between the nervous and the cardiovascular systems. The journal aims to stimulate the development of relevant interdisciplinary and collaborative biomedical research and to foster multidisciplinary efforts in advancing medical practices.

*Hari Manev*

## Figures and Tables

**Figure 1:**
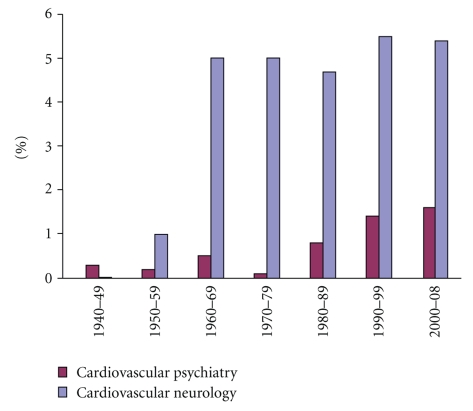
The proportions of retrieved PubMed terms Cardiovascular Psychiatry versus Psychiatry and Cardiovascular Neurology versus Neurology. The PubMed search was performed as described in Table 1. The percentages of items found for *Cardiovascular Psychiatry* and *Cardiovascular Neurology* were calculated based on the corresponding 100% values retrieved for *Psychiatry* and *Neurology*, respectively.

**Table 1:** A number of PubMed items retrieved on January 13, 2009. The terms indicated in the table were searched (http://www.ncbi.nlm.nih.gov/sites/entrez?db=pubmed) for the indicated publication dates (years). No systematic checking was performed for the actual content of individual retrieved items.

Years	Cardiovascular	Neurology	Psychiatry	Cardiovascular neurology	Cardiovascular psychiatry
1940–1949	1712	78	905	0	3
1950–1959	23981	1897	5445	18	13
1960–1969	76270	3641	10080	181	55
1970–1979	151268	4244	14083	216	25
1980–1989	195001	13607	22791	638	188
1990–1999	277125	56631	63399	3152	863
2000–2008	343100	82538	87576	4468	1377

